# Differential presentation and survival of de novo and recurrent metastatic breast cancer over time: 1990–2010

**DOI:** 10.1007/s10549-017-4529-5

**Published:** 2017-10-16

**Authors:** Judith A. Malmgren, Musa Mayer, Mary K. Atwood, Henry G. Kaplan

**Affiliations:** 1 HealthStat Consulting, Inc, 12025 9th Ave NW, Seattle, WA 98177 USA; 2 School of Public Health, University of Washington, Seattle, WA USA; 3 Metastatic Breast Cancer Alliance, New York, NY USA; 4 Swedish Cancer Institute, Seattle, WA USA

**Keywords:** Metastatic breast cancer, Metastases, Survival, Outcomes, Recurrence, De novo, Stage IV, Distant relapse

## Abstract

**Background:**

Differences in de novo (dnMBC) and recurrent metastatic breast cancer (rMBC) presentation and survival over time have not been adequately described.

**Methods:**

A retrospective cohort study, 1990–2010, with follow up through 2015 of dnMBC patients (stage IV at diagnosis) and rMBC patients with subsequent distant metastatic recurrence (stage I–III initial diagnosis) [dnMBC = 247, rMBC = 911)]. Analysis included Chi squared tests of categorical variables, Kaplan–Meier survival estimates, and Cox proportional adjusted hazard ratios (HzR) and 95% confidence intervals (CI). Disease specific survival (DSS) was time from diagnosis or distant recurrence to BC death.

**Results:**

Over time, 1990–1998, 1999–2004, and 2005–2010, dnMBC incidence was constant (3%) and rMBC incidence decreased [18% to 7% (*p* < 0.001)] with no change in dnMBC hormone receptor (HR) or her2-neu (HER2) status but a decrease in rMBC HER2-positive cases and increase in triple negative breast cancer (HR-negative/HER2-negative) (*p* = 0.049). Five-year dnMBC DSS was 44% vs. 21% for rMBC (*p* < 0.001). Five-year dnMBC DSS improved over time [28% to 55% (*p* = 0.008)] and rMBC worsened [23% to 13%, *p* = 0.065)]. Worse DSS was associated with HR-negative status (HzR = 1.63; 1.41, 1.89), rMBC (HzR = 1.88; 1.58, 2.23), older age (70 +) (HzR = 1.88; 1.58, 2.24), > 1 distant metastases (HzR 1.39; 1.20, 1.62), and visceral dominant disease (HzR 1.22; 1.05, 1.43). After 1998, HER2-positive disease was associated with better DSS (HzR = 0.72, 95% CI 0.56, 0.93).

**Conclusions:**

Factors associated with the widening survival gap and non-equivalence between dnMBC and rMBC and decreased rMBC incidence warrant further study.

## Introduction

Metastatic breast cancer (MBC) can present as either stage IV de novo primary breast cancer with distant metastases (dnMBC) (Any T, Any N, M1) or can become metastatic after distant recurrence of initially localized invasive breast cancer (rMBC) (stage I–III) [1]. Estimated new cases of invasive breast cancer in 2017 in the United States (US) are 252,710 of which an estimated 40,610 will die from breast cancer [2]. Long-term evidence comparing dnMBC and rMBC is limited with distant breast cancer recurrence not documented by the US national cancer registry database, Surveillance, Epidemiology, and End Results Program (SEER) [3]. In the US, 6% of all breast cancer cases between 2005 and 2011 or 19,557 cases were stage IV at diagnosis (dnMBC) over the 6-year time span and 5-year relative survival for dnMBC was 26% [4]. It is estimated 80,000 women are alive with rMBC in the US every year with an estimated average life expectancy of 20 months [5].

De novo stage IV and distant recurrent MBC may present with different biology and respond differentially to treatment. Survival differences between dnMBC and rMBC have not been adequately studied, and we hypothesize that differences exist between de novo and recurrent MBC presentation and outcomes. Understanding these differences is important given the utilization of both types of MBC as equivalent entities in some clinical trials. With documented improvement in breast cancer survival over time and ongoing debate regarding the relative impact of screening and treatment, it is also important to characterize factors related to this phenomenon [6, 7]. Our objective is to measure survival changes over time among de novo and recurrent metastatic breast cancer and identify similarities and differences in presentation and diagnosis to model their impact on MBC survival.

## Methods

### Study design

**Fig. 1:**
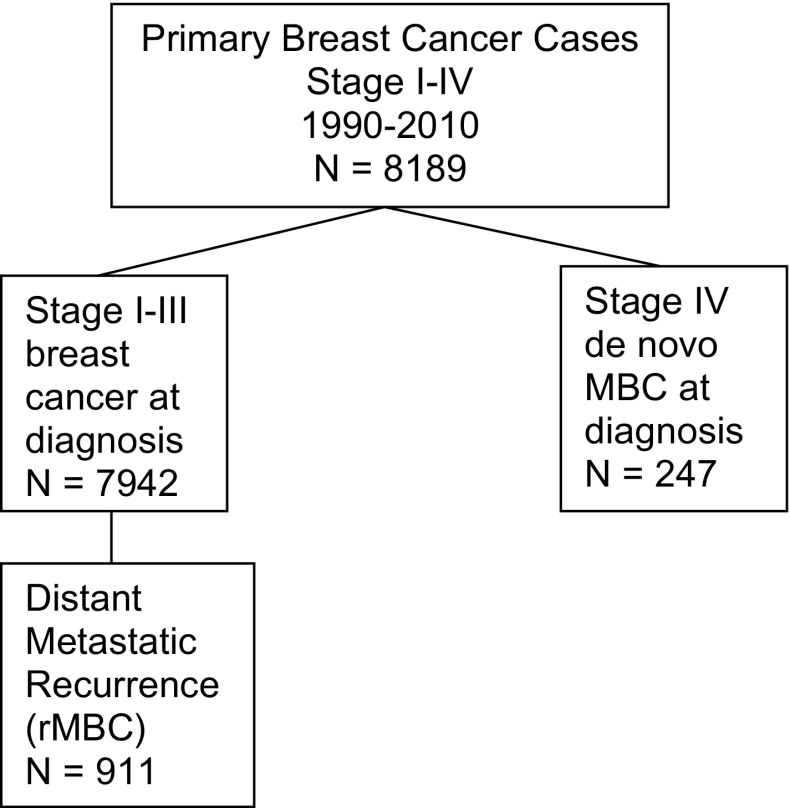
Flow diagram

We conducted a retrospective cohort analysis of de novo and relapsed stage IV MBC patients from prospectively collected data in a dedicated institutional breast cancer registry database, between 1990 and 2010 [invasive BC *N* = 8189, MBC *N* = 1158 (de novo MBC = 247, relapsed MBC = 911)] (Fig. 1). Primary de novo stage IV MBC was identified at diagnosis using American Joint Committee on Cancer (AJCC) 7 diagnostic criteria (Any T, Any N, M1) [1]. Relapsed MBC (rMBC) cases were identified by annual follow up of primary stage I-III patients for distant metastatic recurrence. Date and site(s) of distant recurrence are documented in the registry. Additional follow up and review of death certificate information were obtained to verify cause of death. Cases that were alive with cancer status unknown (*n* = 81), died with unknown cancer status (*n* = 59), those untreated due to age or preference (*n* = 7), or lost to follow up before 2 years post diagnosis (*n* = 33) were excluded from the analysis.

Our institutional breast cancer registry was created in 1990 and contains detailed information on diagnosis, pathology, staging, surgery, chemotherapy, radiation therapy, tumor markers, vital status at follow up including cause-specific death. Incident BC cases are entered at time of diagnosis into the registry. Patient vital and disease status including date and site of recurrence and date and cause of death is collected prospectively through annual updates by a certified cancer registrar complete through 2015 for this cohort. Information is obtained from (1) electronic chart review, (2) IRB approved physician directed follow up letter, (3) the institution’s cancer registry, and (4) SEER Seattle-Puget Sound Registry [8].

Distant disease recurrence for dnMBC and rMBC was restricted to the site(s) at first presentation of distant disease and excluded sites of subsequent disease progression. Dominant distant recurrence site was (1) soft tissue if distant lymph nodes or skin metastases but not bone or visceral, (2) bone if bone metastases with or without soft tissue or visceral, and (3) visceral if metastases to organs with or without bone or soft tissue involvement. We defined hormone receptor positive patients as estrogen and/or progesterone receptor positive (HR positive) and HR-negative if negative for both. Breast cancer detection methods were recorded at time of diagnosis from the chart notes and were the following: (1) patient detected: lump or abnormality discovered by the patient (symptomatic); (2) physician detected: lump or abnormality discovered during routine physical examination (symptomatic); or (3) mammography detected: lump or breast abnormality discovered by a non-diagnostic mammogram.

Cases were considered rMBC if distant recurrence occurred 3 months or more post initial diagnosis; a single patient with distant recurrence at 2 months was included after confirmed negative for metastases by scans and imaging. Distant disease-free interval (DFI) was calculated as time from primary BC diagnosis to distant recurrence (metastatic disease diagnosis) for rMBC patients. We modeled disease specific survival (DSS) interval as time from diagnosis date to breast cancer death for de novo MBC and time from first distant relapse to breast cancer death for rMBC patients [9].

Covariates affecting initial and subsequent choice of treatment including age, hormone receptor, and diagnosis time period were selected for inclusion in the Cox proportional hazards model a priori. Her2/neu (HER2) testing and taxane therapy for HER2+ patients became standard of care at our institution in 1999. A second model was run on the subset of cases diagnosed from 1999 to 2010 with the original model and HER2 test results.

The time periods, 1990–1998, 1999–2004, 2005–2010, used for our NCCN guideline compliant institutional cohort were selected by coincidental timing of changes in systemic therapy for invasive and metastatic breast cancer including hormone therapy, trastuzumab, and taxanes and neoadjuvant therapy administration [10]. Appropriateness of time period designation was confirmed by measurement of statistically significant changes in initial systemic therapy and neoadjuvant therapy for invasive stage I–III BC and systemic therapy for stage IV MBC in our cohort coincident with the three time periods (Table 1). Additionally, treatment was not included in the model as it is intermediate in the pathway to outcome and predicated by presentation characteristics and diagnosis year standard of care. Therefore, time period was included as the proxy for treatment in the model based on the assumption and evidence that NCCN standard of care protocols per time period at diagnosis were utilized.

**Table 1:** Change in systemic therapy 1990–2010: stage I–IV (n = 8189)

	1990–1998	1999–2004	2005–2010	*p* Value
	*N* (%)	*N* (%)	*N* (%)	
Hormone therapy for hormone receptor positive patients				
Stage I–III (*n* = 6334)	1353 (72%)	1753 (90%)	2328 (93%)	< 0.001
Stage IV (dnMBC) (*n* = 177)	48 (30%)	42 (26%)	72 (44%)	0.317
Adjuvant chemotherapy patients: Stage I–III (*n* = 4235)	1270 (52%)	1295 (53%)	1670 (55%)	0.124
Taxane therapy				
Stage I–III	180 (14%)	664 (51%)	1156 (69%)	< 0.001
Stage IV (dnMBC) (*n* = 163)	11 (17%)	16 (25%)	37 (58%)	0.002
Trastuzumab therapy for HER2 positive patients				
Stage I–III (*n* = 846)	–	86 (29%)	359 (89%)	< 0.001
Stage IV (dnMBC) (*n* = 43)	–	6 (22%)	21 (100%)	< 0.001
Neoadjuvant therapy for chemotherapy patients				
Stage I–III (*n* = 4247)	155 (27%)	160 (28%)	260 (45%)	0.011

### Statistical analysis

Tests of statistical significance, mean comparisons (F statistic), Chi square tests (Pearson Chi square), Kaplan–Meier estimation of survival (log rank tests), and multivariable Cox proportional hazards models were used to estimate adjusted hazard ratios (HzR) and corresponding 95% confidence intervals (CI). Proportional hazards assumptions were evaluated by (1) testing for interaction between time period and the logarithm of follow up time and (2) graphically by plotting log–log KM versus log-time. No evidence of violation of the proportionality assumption was found. All *p*-values were 2-sided and analyses were conducted using SPSS v.24 [11].

### Role of funding source

The funding sources, The Kaplan Cancer Research Fund and the Metastatic Breast Cancer Alliance, did not have any role in the study design, collection, analysis or interpretation of data, the writing of the article or the decision to submit the paper for publication.

### IRB approval

This project and the registry the data was drawn from both received IRB approval prior to the collection and use of the data. IRB approved and HIPPAA compliant methods were used for data collection, storage and analysis with de-identified data.

## Results

### Cohort characteristics

In our institutional cohort registry of first primary invasive breast cancer from 1990 to 2010 (*n* = 8189), 49% of cases were stage I, 34% stage II, 14% stage III, and 3% stage IV (dnMBC) (*n* = 247). Fourteen percent of the entire cohort were non-white (Asian, Hispanic, Black, Native American, other). Of the 7942 stage I–III invasive breast cancer cases, 11.5% developed distant metastatic recurrence (rMBC) (*n* = 911). dnMBC and rMBC were significantly younger than the stage I–III non-MBC cases [mean age in years dnMBC = 55.3, rMBC = 53.9, stage I–III = 57.6, *p* < 0.001]. The majority of cases that were metastatic at diagnosis (dnMBC) or localized at diagnosis and became metastatic (rMBC), 91% and 74%, respectively, were detected by symptoms (patient or medical professional) whereas cases that were stage I-III at diagnosis and did not become metastatic were more often mammography detected (54%) (*p* < 0.001).

At initial presentation, 57% of dnMBC patients had surgical excision of their breast cancer by either lumpectomy or mastectomy and 67% had adjuvant chemotherapy. At initial presentation of stage I–III breast cancer, all rMBC patients had surgery and 78% had adjuvant chemotherapy. Ninety-two percent of hormone receptor positive dnMBC (92%) and 85% of rMBC at primary stage I–III diagnosis had hormone therapy as initial breast cancer treatment.

#### dnMBC/rMBC comparison

**Fig. 2:**
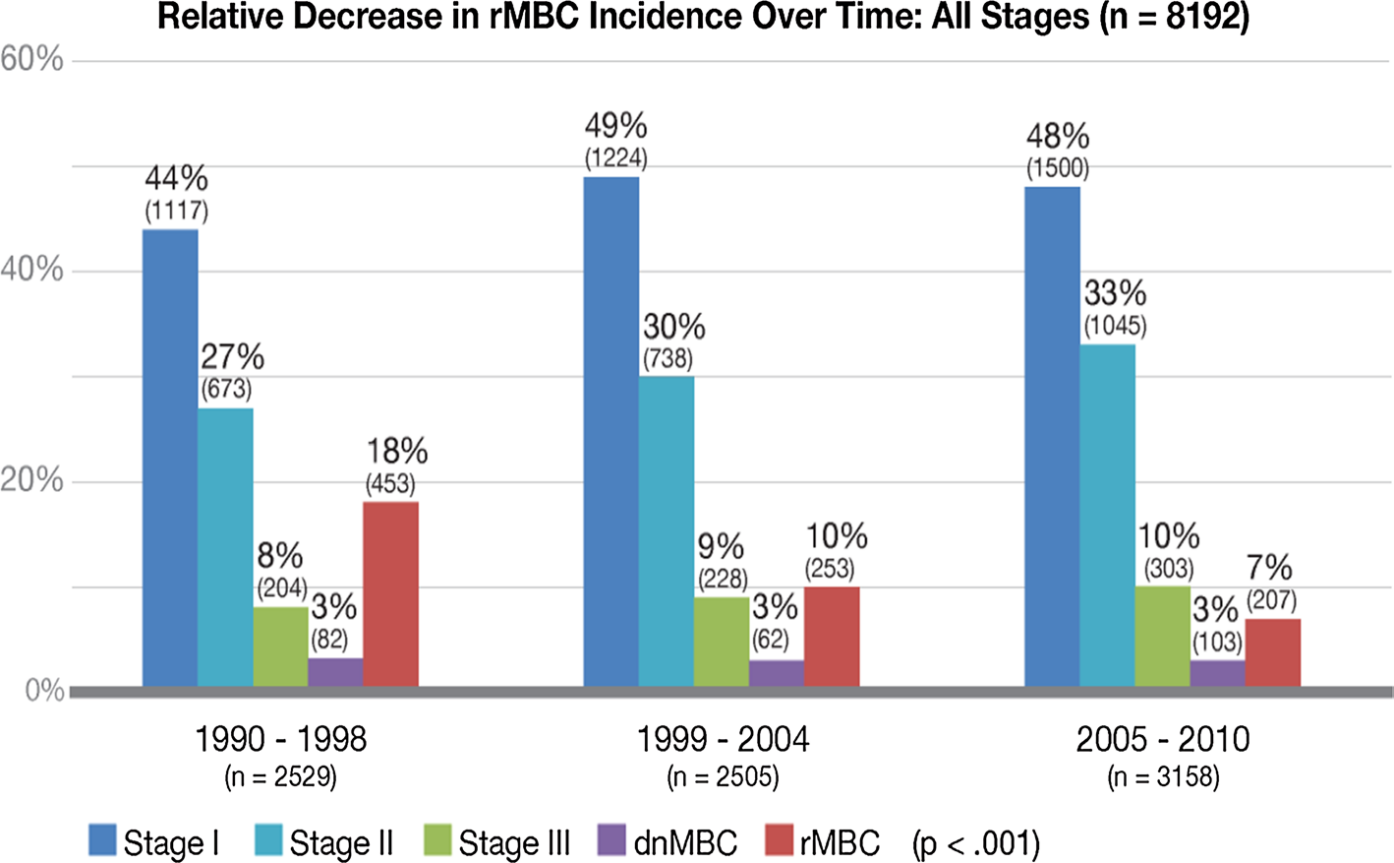
Relative decrease in rMBC incidence over time: All stages (*n* = 8192)

**Fig. 3:**
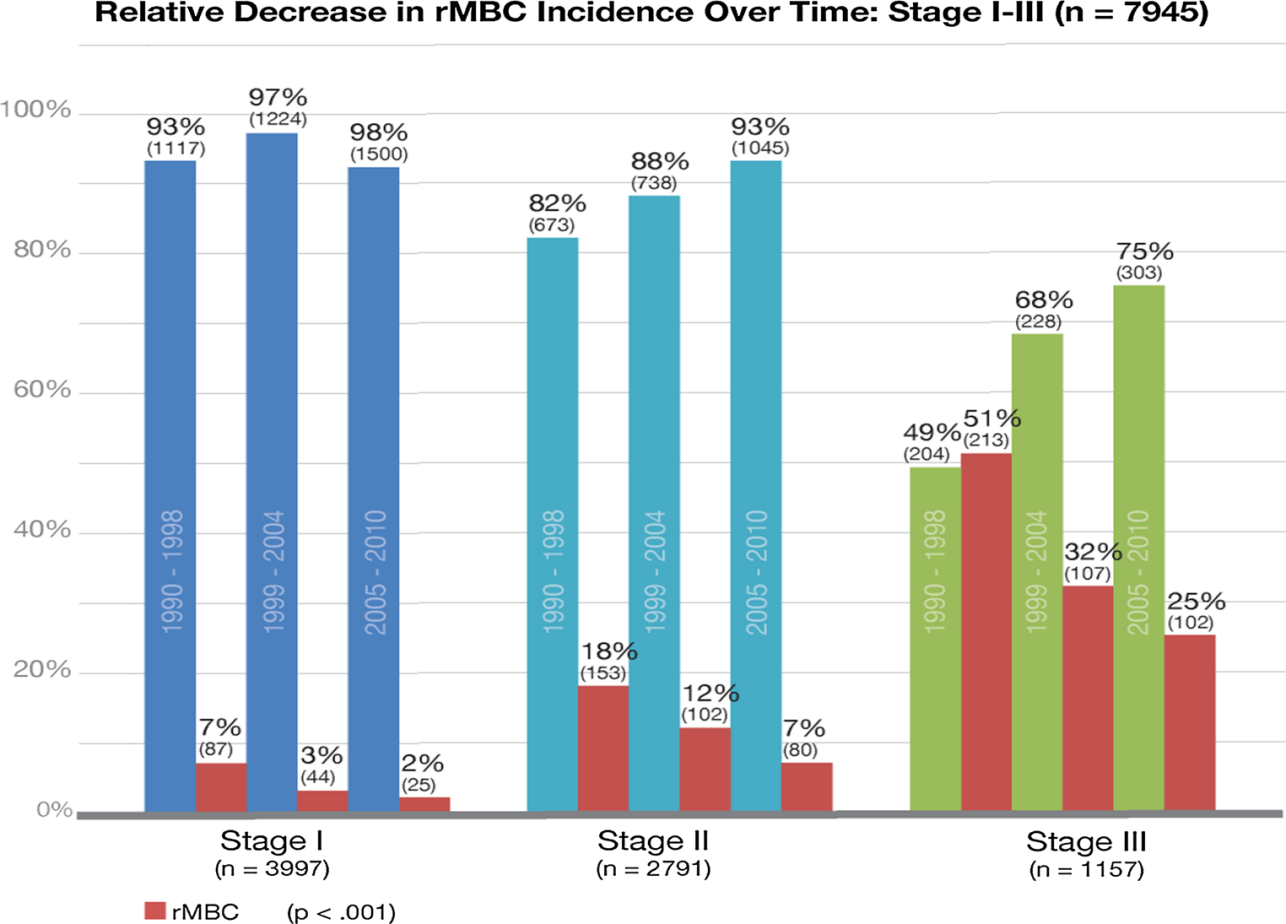
Relative decrease in rMBC incidence over time: Stage I–III (*n* = 7945)

Incidence of dnMBC did not change over time and remained constant at 3% per year. HR and HER2 status remained the same over the entire period among dnMBC cases. rMBC incidence decreased by more than half over time [1990–1998 = 18% (*n* = 453), 1999–2004 = 10% (*n* = 253), 2005–2010 = 7% (*n* = 207) (*p* < 0.001] (Fig. 2). The reduction in rMBC incidence was consistent across all initial diagnosis stages [stage I: 7% to 2%; stage II: 19% to 7%; stage III: BC 51% to 25% (*p* < .001)] (Fig. 3). With the decline in rMBC incidence over time, the ratio of rMBC to dnMBC cases decreased from 5.5:1 in 1990–1999 to 4:1 in 2000–2004 to 2:1 in 2005–2010.

dnMBC and rMBC cases did not differ by age at initial diagnosis [dnMBC mean age = 55.3 years, range 24–94; rMBC mean age = 53.85, range 23–93; *F* statistic = 2.301, *p* = 0.130]. dnMBC were more often non-white [dnMBC = 20% vs. rMBC = 12% (*p* = 0.003)] (Table 2). Hormone receptor and her2-neu status individually did not differ between dnMBC and rMBC patients (Table 2). rMBC cases were more often triple negative subtype (HR−/HER2−) [23% vs. 11%, *p* = 0.005] (Table 2). The majority of both dnMBC and rMBC cases were ductal. dnMBC cancers was more likely to have less common histologic types classified as ‘other’ including adenocarcinoma, carcinoma NOS, metaplastic, colloid/mucinous, and tubular (Table 2). Significantly more of the dnMBC cases had high histologic grade [88% vs. 78%, *p* = 0.002].

**Table 2:** dnMBC and rMBC descriptive comparisons (*n* = 1158)

	DnMBC	RMBC	*p* Value
	(*n* = 247)	(*n* = 911)	
	*N* (%)	*N* (%)	
Age			
20–39	32 (13%)	132 (14%)	0.758
40–49	56 (23%)	235 (26%)	
50–59	69 (28%)	242 (27%)	
60–69	50 (20%)	162 (18%)	
70+	40 (16%)	140 (15%)	
Race			
White	198 (80%)	798 (88%)	0.003
Non-White	49 (20%)	113 (12%)	
Diagnosis year of first primary breast cancer			
1990–1998	82 (33%)	451 (50%)	< 0.001
1999–2004	62 (25%)	253 (28%)	
2005–2010	103 (42%)	207 (23%)	
Initial breast tumor detection method			
By patient or physician (symptomatic)	201 (91%)	660 (74%)	< 0.001
By mammography	21 (9%)	236 (26%)	
Hormone receptor status at initial diagnosis (*n* = 1137)			
HR+	182 (77%)	658 (72%)	0.189
Her2/neu status at initial diagnosis (*n* = 613)^a^			
Her2+ (HR− or HR+)	35 (22%)	74 (16%)	0.132
HR/Her2 status at initial diagnosis (*n* = 613)^a^			
HR+/HER2−	109 (68%)	274 (61%)	0.005
HR+/HER2+	17 (11%)	47 (10%)	
HR-/HER2−	18 (11%)	104 (23%)	
HR-/HER2+	17 (11%)	27 (6%)	
Histologic type of initial primary breast tumor			
Ductal	183 (74%)	738 (81%)	0.016
Lobular	33 (13%)	104 (12%)	
Lobular/ductal mixed	10 (4%)	36 (4%)	
Other cancer	21 (9%)	33 (4%)	
Nuclear grade of initial primary breast tumor			
Low	7 (3%)	36 (4%)	0.815
Intermediate	78 (37%)	306 (36%)	
High	124 (59%)	508 (60%)	
Histologic grade of initial primary breast tumor			
Low	0	19 (2%)	0.002
Intermediate	25 (12%)	169 (20%)	
High	185 (88%)	661 (78%)	
Tumor size (mean, range and significance of F statistic)	5.87 cm (0.5–20 cm)	3.60 cm (0.1–18 cm)	< 0.001
Number of positive lymph nodes (mean, range and significance of F statistic)	7.11 (0-33)	4.52 (0-36)	< 0.001
Dominant site of distant metastases			
Bone	102 (42%)	303 (33%)	0.001
Visceral	107 (44%)	513 (56%)	
Soft tissue	37 (15%)	93 (10%)	
Distant metastatic sites at MBC diagnosis^b^			
Bone	144 (58%)	494 (54%)	
Liver	52 (21%)	213 (23%)	
Lung	52 (21%)	255 (28%)	
Brain	3 (1%)	75 (8%)	
Skin	7 (3%)	38 (4%)	
Lymph nodes	82 (33%)	179 (20%)	
Number of distant metastatic sites at MBC diagnosis			
1	166 (68%)	575 (63%)	0.196
2+	80 (32%)	335 (37%)	
Mean survival years post MBC diagnosis (years)	5.03	2.81	< 0.001
Vital Status			
Alive NED	27 (11%)	30 (3%)	< 0.001
Alive with disease	26 (11%)	118 (13%)	
Died NED	2 (0.8%)	8 (0.9%)	
Died with disease	191 (77%)	749 (82%)	

^a^Trastuzumab FDA approval 1998, consistent Her2/neu testing began in 1999

^b^Does not add to 100% as cases may have multiple metastatic sites, no Chi square calculated

Number of metastatic sites, 1 vs. 2 or more, did not differ between dnMBC and rMBC (Table 2). By individual site, bone was the most common metastatic site in both types of MBC [dnMBC: *n* = 144 (58%), rMBC: *n* = 494 (54%)]. Using a hierarchical measurement of metastases by dominant site [(1) visceral (lung, liver or brain), (2) bone, or (3) soft tissue)], visceral was most common among both dnMBC and rMBC [dnMBC = 44%, rMBC = 56%]. At first presentation of metastatic disease, 1% (3/246) of dnMBC had brain metastases vs. 8% (75/911) of rMBC. Significantly more dnMBC cases were alive with no evidence of disease (NED) at 5 or more years follow up [11% vs. 3%, *p* < 0.001]. (Table 2).

#### rMBC

For rMBC cases, distribution by age and stage at initial diagnosis did not change over time (Table 3). Number of hormone receptor positive cases declined over time but not significantly. Post 1998, percent HER2 positive patients declined over time and triple negative (HR−/her2−) rMBC cases increased (*p* = 0.044) (Table 3). Percent of patients with bone dominant metastatic site declined from 37 to 21% over time (*p* < 0.001).

**Table 3:** rMBC characteristic comparisons by diagnosis year (n = 911)

	1990–2010	1990–1998	1999–2004	2005-–2010	*p* Value
	*N* (%)	*N* (%)	*N* (%)	*N* (%)	
Number of patients	911 (100%)	452 (50%)	253 (28%)	207 (23%)	
Age at initial diagnosis (years)	54 (23-93)	53.13	54.91	54.24	0.218
Stage at initial diagnosis					
I	156 (17%)	87 (19%)	44 (17%)	25 (12%)	0.096
II	334 (37%)	152 (34%)	102 (40%)	80 (39%)	
III	421 (46%)	212 (47%)	107 (42%)	102 (49%)	
Hormone receptor status					
Positive	658 (72%)	331 (75%)	184 (73%)	143 (69%)	0.313
Her2/neu status^a^ (*n* = 452)					
Positive	74 (16%)		48 (20%)	26 (13%)	0.044
HR/HER2 status^a^ (*n* = 452)					
HR+/HER2−	274 (61%)		150 (61%)	124 (60%)	0.041
HR+/HER2+	47 (10%)		28 (11%)	19 (9%)	
HR−/HER2−	104 (23%)		47 (19%)	57 (28%)	
HR−/HER2+	27 (6%)		20 (8%)	7 (3%)	
Dominant site of distant metastases					
Bone	303 (33%)	168 (37%)	92 (37%)	43 (21%)	< 0.001
Visceral (lung, liver, brain)	513 (56%)	232 (52%)	140 (56%)	141 (68%)	
Soft tissue (lymph nodes, skin)	93 (10%)	50 (11%)	20 (8%)	23 (11%)	
Disease-free interval (years)	4.89 (0.25–20.46)	5.47 (0.46–20.46)	4.90 (0.39–16.82)	3.62 (0.17–10.28)	< 0.001

^a^Post 1998 cases only, after her2-neu testing became standard for all breast cancer patients

Mean follow up to distant recurrence for rMBC cases was 4.89 years [median 3.64 years, range = 0.17, 20.46 years]. Disease-free interval (DFI) from initial diagnosis date to distant recurrence date for rMBC cases decreased over time from 5.47 years to 3.62 years [1990–1998 = 5.47 years (range = 0.46–20.46); 1999–2004 = 4.90 years (range = 0.39–16.82); 2005–2010 = 3.62 years (range = 0.17–10.28) (*p* < 0.001] (Table 3). Hormone receptor negative invasive breast cancer cases were most likely to have distant recurrence in the first 5 years post initial diagnosis (88%) [median DFI = 2.6 years] while hormone receptor positive rMBC cases had longer time to distant recurrence with half before and half after 5 years post initial diagnosis [median DFI = 5.0 years] (*p* < 0.001).

#### Survival analysis

**Fig. 4:**
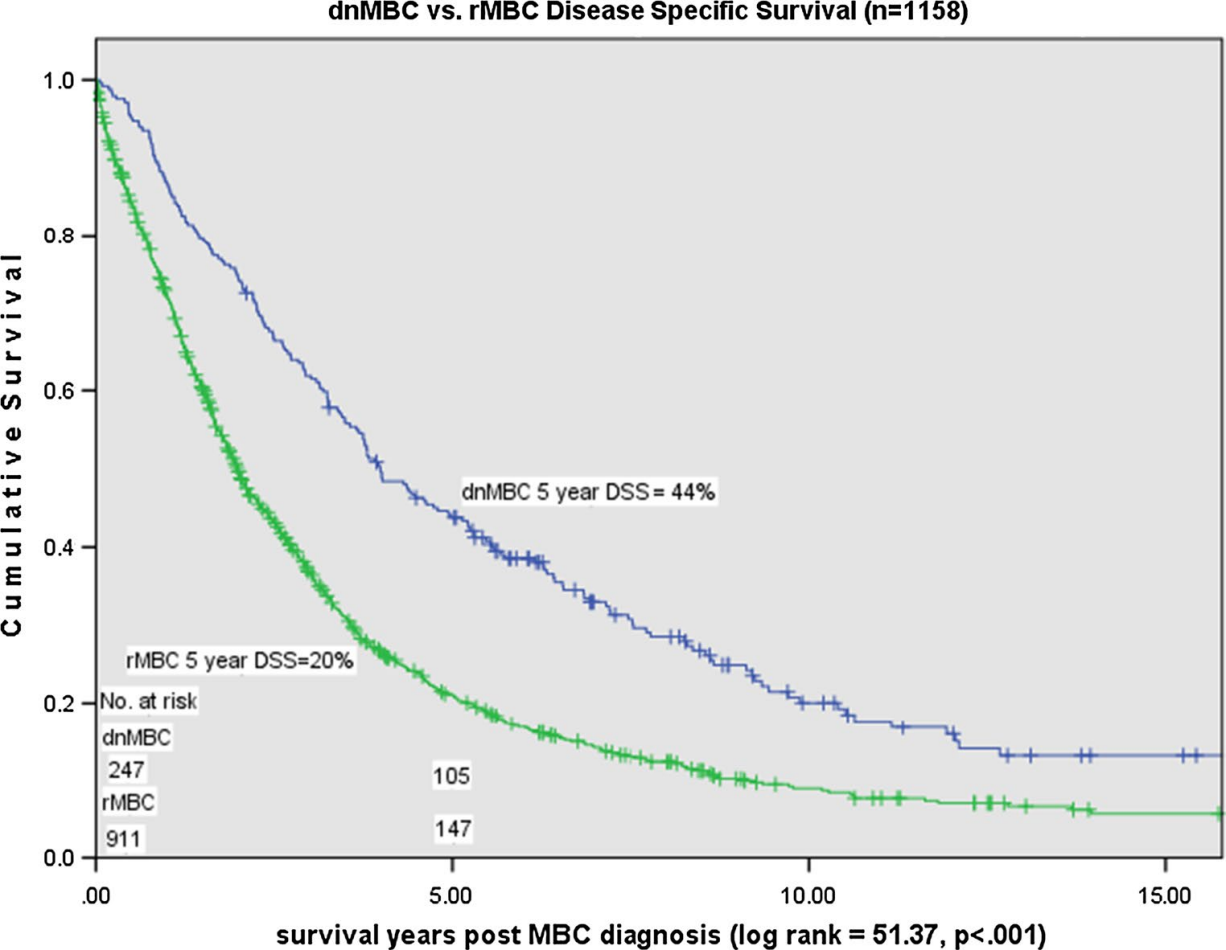
dnMBC and rMBC comparative disease specific survival

**Fig. 5:**
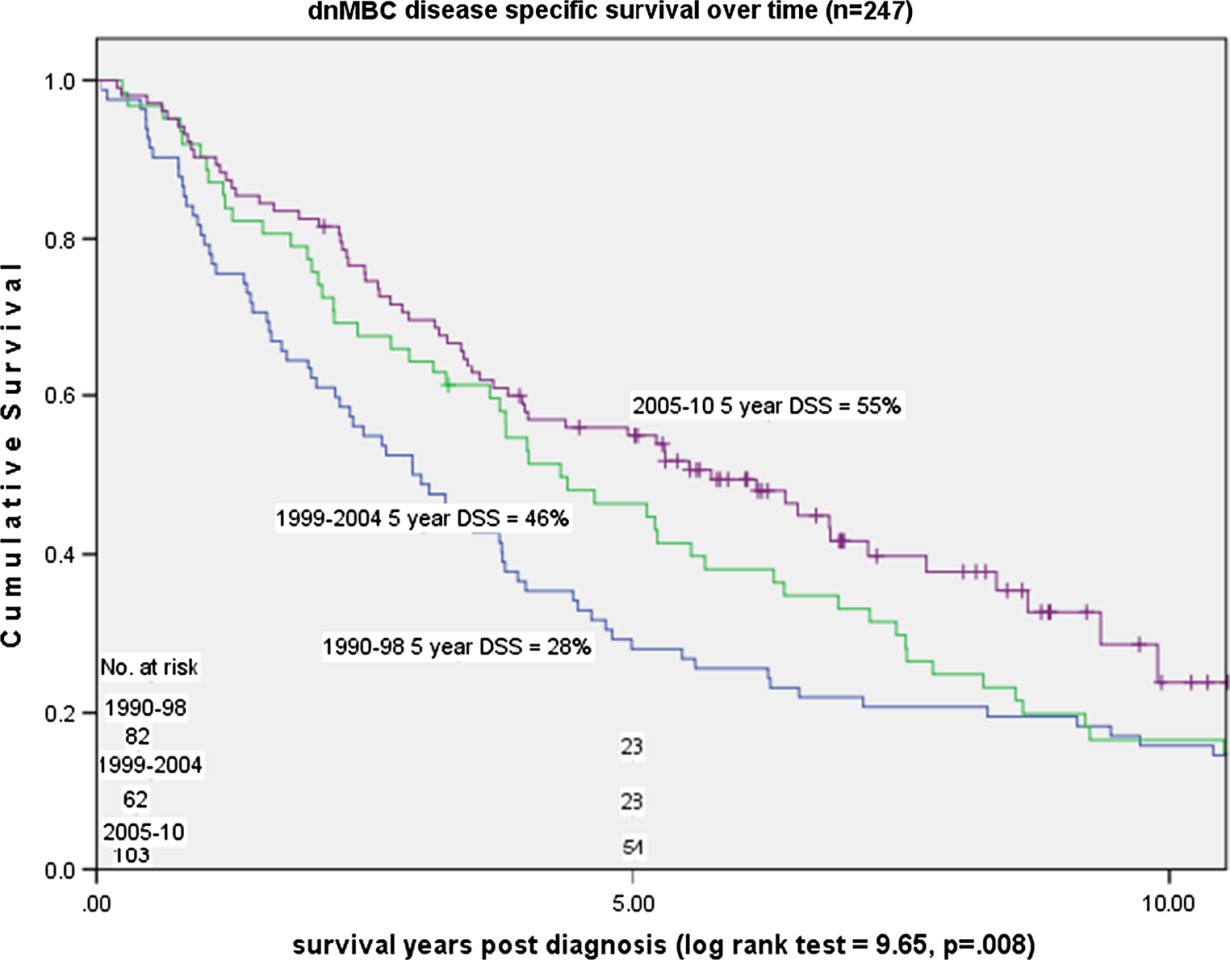
dnMBC change in disease specific survival over time: 1990–2010

**Fig. 6:**
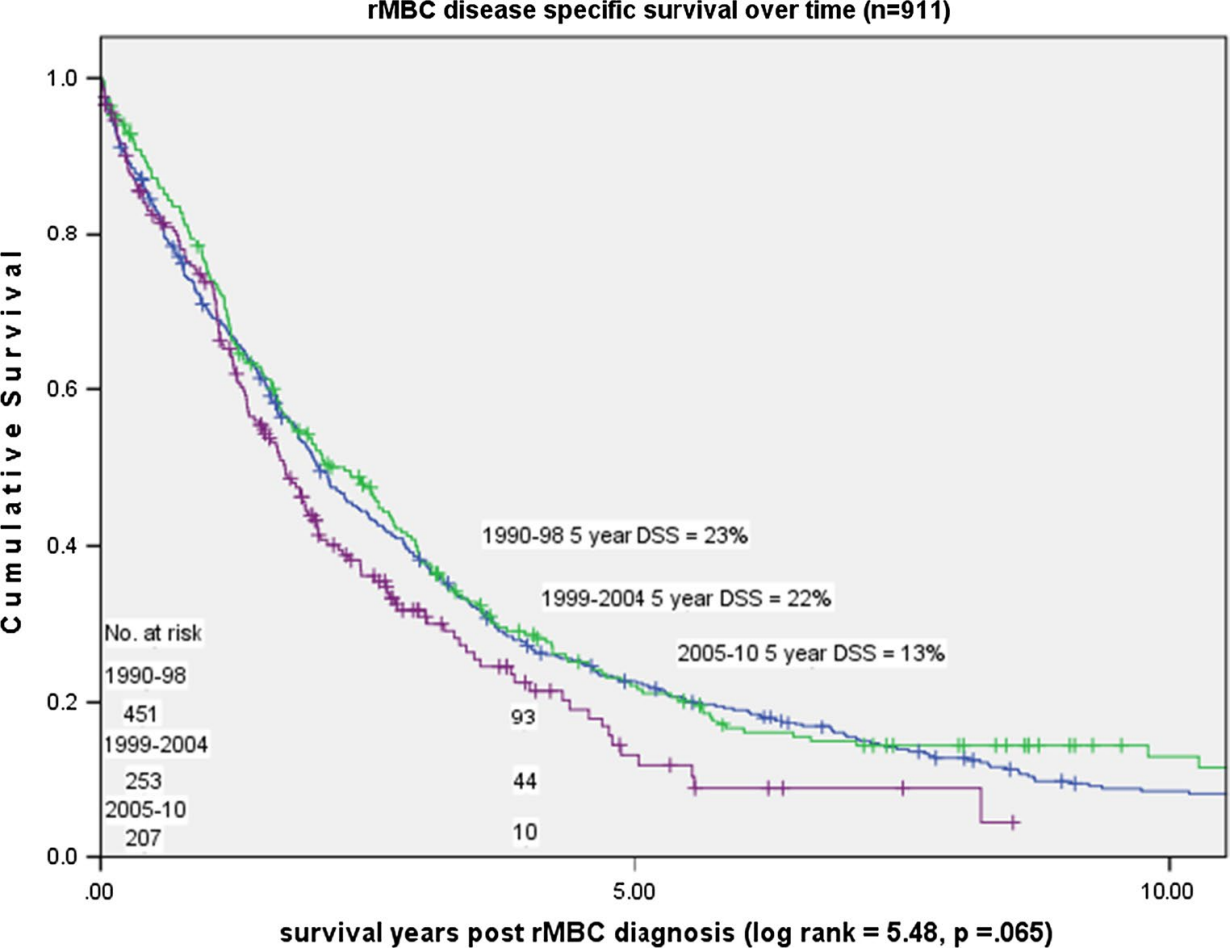
rMBC change in disease specific survival over time: 1990–2010

Median survival post MBC diagnosis was 3.92 years (mean 5.03 years) for dnMBC and 1.83 years (mean 2.81 years) for rMBC (*p* < 0.001). Overall years, 5-year DSS was 44% for dnMBC cases and 20% for rMBC cases (*p* < 0.001) (Fig. 4). Over time, 5-year DSS improved for dnMBC cases from 28 to 55% (*p* = 0.008) but declined for rMBC cases from 23 to 13% (*p* = 0.07) (Figs. 5, 6).

In multivariable models with adjustment for race and diagnosis year, worse DSS was associated with negative hormone receptor status (HzR = 1.66, 95% CI 1.42, 1.93), rMBC vs. dnMBC diagnosis (HzR = 1.82, 95% CI 1.53, 2.16), > 1 metastatic site at diagnosis (HzR = 1.39, 95% CI = 1.20, 1.93), visceral dominant site of metastases compared to bone (HzR = 1.22, 95% CI 1.05, 1.43) and age 70 or greater at initial diagnosis (HzR = 1.97, 95% CI 1.66, 2.34) (*n* = 1158) (Table 4). In the same model including only cases diagnosed in 1999 or later (*n* = 625), HER2 positive status was associated with better survival (HzR = 0.68, 95% CI 0.52, 0.88).

**Table 4:** Cox proportional hazards model (*n* = 1158)

	HzR (95% CI)	*p* Value
Negative hormone receptor status vs. positive	1.66 (1.42, 1.93)	< 0.001
Age ≥ 70 years vs. < 70 years	1.97 (1.66, 2.34)	< 0.001
rMBC vs. dnMBC	1.82 (1.53, 2.16)	< 0.001
≥ 2 metastatic sites at diagnosis vs. 1	1.39 (1.20, 1.62)	< 0.001
Visceral dominant distant metastatic site vs. bone	1.22 (1.05, 1.43)	0.012

Adjusted for race white/non-white and diagnosis year time period

*CI* confidence intervals, *rMBC* recurrent metastatic breast cancer*, dnMBC* de novo metastatic breast cancer*, HzR* hazard ratio, *Visceral* lung, liver, brain

## Discussion

In our cohort, we observed significant improvement in dnMBC survival but a reduction in rMBC incidence with worse survival over time using cohorts coincident with major changes in treatment for invasive and de novo stage IV BC (1990–1998, 1999–2004, 2005–2010). Triple negative subtype increased and HER2+ subtype decreased over time among rMBC cases with a concurrent shortening of disease-free interval from time of initial invasive breast cancer diagnosis to incidence of distant recurrence and rMBC diagnosis. dnMBC and rMBC had similarities but statistically significant differences in both presenting characteristics and outcomes. Both types of MBC cases were more often younger than age 70 and patient/medical professional detected compared to stage I–III non-metastatic breast cancer. Both dnMBC and rMBC were primarily high histologic grade at initial diagnosis. dnMBC dominant metastatic disease site was more likely bone than rMBC which were more often visceral.

dnMBC incidence and presentation did not change over time. rMBC incidence declined from 18% in 1990–1998 to 7% in the last time period, 2005–2010. rMBC: dnMBC ratio declined over time from 5:1 to 2:1. The survival difference increased from 5% in 1990–1998 to 24% in 1999–2004 and 42% in 2005–2010. rMBC bone dominant distant recurrence declined over time with a relative increase in visceral and soft tissue dominant disease. Both dnMBC and rMBC had better survival for single site metastatic disease (oligometastatic). Time to relapse for hormone receptor negative rMBC was significantly shorter, more often less than 5 years, than for hormone receptor positive rMBC.

From our modeling study, a number of MBC presentation and disease characteristics are probable contributors to the difference in dnMBC and rMBC survival and declining rMBC incidence. De novo MBC has a number of characteristics which may confer a survival advantage over recurrent MBC. These are the following, (1) more often a single metastatic site, (2) more likely hormone receptor positive with single bone metastases which can be treated successfully with hormonal therapy, (3) no treatment limitations from chemotherapy resistance (treatment naïve), (4) trastuzumab treatment for HER2+ disease.

rMBC cases declined over time due to both better primary breast cancer treatment over time (hormone therapy, taxanes, and trastuzumab) and improved screening with detection of cancer at an earlier more treatable stage (reduction of stage II and III). The rMBC cohort changed over time to more triple negative and fewer HER2+ cases after the introduction of trastuzumab in 1999. The rMBC survival disadvantage may be due to fewer distant recurrence cases with a more difficult treatment profile and possible chemotherapy resistance from aggressive first line treatment.

In our study, 5-year breast cancer survival was significantly better overall for de novo MBC and improved continuously over time. A recent study estimates de novo MBC 5- year relative survival has improved from 18 to 36% over time among younger women [12]. In a recent Canadian study, a similar dnMBC/rMBC survival difference was observed with dnMBC 5-year survival 24% and rMBC survival 12% in a single time period (2001–2009) [13]. Their model found increased mortality hazard associated with older age and relapsed versus de novo MBC. In a model using only de novo MBC, Leone et al. observed increased mortality hazard with older age and triple negative status [14]. Vaz-Luis et al. also observed dnMBC survival improvement in a shorter survival time which may skew data to triple negative patient deaths and patients ineligible for standard treatment [15].

In our model, we found bone dominant site associated with better survival which may account for a portion of the survival difference as dnMBC is more often bone dominant than rMBC. Better clinical outcomes have been observed among bone-only MBC patients [16]. Superior long-term survival in oligometastatic disease has been observed with greater than one metastatic site identified as an adverse risk factor [17–19].

In 2000, Sir Richard Peto noted a marked 25% decline in BC deaths in the UK and USA for 20–69 year old patients related to early detection by mammography and hormonal and cytotoxic adjuvant treatment changes [6]. Meta-analysis of randomized trials has found mortality reduction related to treatment changes, primarily taxane-plus-anthracycline and higher-cumulative dose anthracycline-based regimens [20]. In a large longitudinal study of MBC in Sweden, survival improvement was observed in a more recent time period (2000–2004) for patients 60 years or younger [21]. A Canadian MBC survival study found population-based improvements in the most recent cohort related to the release of new systemic agents for MBC including trastuzumab and taxanes [22].

In a previous study of invasive breast cancer at our institution, mammography detection, hormone therapy, and taxane-containing chemotherapy were associated with decreased hazard of mortality over time [7]. In a study by Wu et al., identification of breast cancer by symptoms as opposed to a mammogram was an independent predictor of recurrence [23]. The majority of both rMBC and dnMBC patients in the current study were symptomatically detected either clinically or by the patient in a time period when mammography screening was readily available and mammography detected breast cancer increased [24].

The observed reduction in both HER2+ and HR+ rMBC cases over time indicates increasing success of initial targeted therapy with trastuzumab and hormone therapy [25–27]. Decreased distant recurrence among HER2+ patients is consistent with reported results of improved long-term outcomes after neoadjuvant/adjuvant treatment with HER2 targeted therapy [28–30]. Improvements in early disease treatment targeted at hormone receptor and HER2 positive disease has reduced overall distant relapse rates but left a remainder of rMBC cases with more aggressive disease and fewer treatment options.

A strength of our study is the meticulous patient follow up for recurrence and vital status by a dedicated registrar. Our modeling did not include time to distant recurrence as it is an intermediate outcome and would interfere with interpretation of the presenting characteristics’ relationship to survival. Differential time to recurrence by HR status may skew incidence of TNBC versus HR+ rMBC as the last time period (2005–2010) has shorter follow up than the previous time periods [31]. Inclusion of diagnostic year interval in the Cox model adjusts for differential follow up time in later years and treatment changes over time. We are only able to analyze outcomes based on HR/HER2 status after 1998.

dnMBC is inherently treatment naive which may confer a survival advantage with better response to treatment and decreased likelihood of chemo-resistance [32, 33]. rMBC cases may be more likely to have or develop intra-tumor heterogeneity after primary exposure to chemotherapy which fosters subsequent therapeutic failure for metastatic disease [34]. dnMBC patients may represent a less complex disease type than breast cancer patients who present with localized disease and subsequently develop distant recurrence (rMBC) [35]. Future studies of tumor genomics to study differential response between dnMBC and rMBC receiving the same therapy may help to understand these differences.

Our observation of a significantly greater survival improvement for de novo MBC than that seen in other studies suggests a need for demographic and treatment comparisons in other populations to explain and understand lesser outcomes. Characterization of dnMBC and rMBC with worse survival can be used to focus research on breast cancer subtypes that continue to have poor outcomes [36]. Expansion of national registry data to capture distant recurrence to track recurrent disease survival would make these types of studies possible. MBC treatment, population, and cohort studies may need to include separate evaluation of de novo and recurrent MBC as their presentation and outcomes indicate a possible differential response to therapy.

Our study provides a community-based confirmation of HER2-directed therapy effectiveness to support expanded access to adjuvant HER2-directed treatment. Current use of targeted therapy using single tumor biopsies may be adequate for only a portion of MBC treatment planning. Research using tumor sequencing and patient-derived xenografts to study biologic evolution of breast cancer clones and complex tumor response to new treatments may help the hardest to treat achieve better outcomes [37].

The advent of effective targeted therapy for hormone receptor positive disease, HER2 positive disease and taxane therapy in the adjuvant/neoadjuvant setting coincide with improved dnMBC survival and decline in rMBC incidence. Patients with recurrent metastatic disease who are ineligible for specialized treatment have a poor outlook. Tailored care of patients most at risk for distant disease recurrence and the expansion of up to date treatment use may be an opportunity to improve outcomes.
